# Dynamically Adjusting Borophene-Based Plasmon-Induced Transparency in a Polymer-Separated Hybrid System for Broadband-Tunable Sensing

**DOI:** 10.3390/polym15143060

**Published:** 2023-07-16

**Authors:** Kunpeng Xiao, Junming Li, Hui Zhang, Huan Jiang, Weiren Zhao

**Affiliations:** 1 School of Physics and Optoelectronic Engineering, Guangdong University of Technology, Guangzhou 510006, China; 2 Guangdong Provincial Key Laboratory of Information Photonics Technology, Guangdong University of Technology, Guangzhou 510006, China

**Keywords:** borophene, polymethyl methacrylate, PIT, sensor

## Abstract

Borophene, an emerging two-dimensional (2D) material platform, is capable of supporting highly confined plasmonic modes in the visible and near-infrared wavebands. This provides a novel building block for light manipulation at the deep subwavelength scale, thus making it well-suited for designing ultracompact optical devices. Here, we theoretically explore a borophene-based plasmonic hybrid system comprising a continuous borophene monolayer (CBM) and sodium nanostrip gratings (SNGs), separated by a polymer spacer layer. In such a structure, a dynamically tunable plasmon-induced transparency (PIT) effect can be achieved by strongly coupling dark and bright plasmonic modes, while actively controlling borophene. Here, the bright mode is generated through the localized plasmon resonance of SNGs when directly excited by TM-polarized incident light. Meanwhile, the dark mode corresponds to a propagating borophene surface plasmon (BSP) mode in the CBM waveguide, which cannot be directly excited, but requires phase matching with the assistance of SNGs. The thickness of the polymer layer has a significant impact on the coupling strength of the two modes. Owing to the BSP mode, highly sensitive to variations in the ambient refractive index (RI), this borophene-based hybrid system exhibits a good RI-sensing performance (643.8 nm/RIU) associated with a wide range of dynamically adjustable wavebands (1420–2150 nm) by tuning the electron density of borophene. This work offers a novel concept for designing active plasmonic sensors dependent on electrically gating borophene, which has promising applications in next-generation point-of-care (PoC) biomedical diagnostic techniques.

## 1. Introduction

Surface plasmons (SPs) can couple electromagnetic waves strongly to the metal surface at subwavelength scales and thereby greatly enhance light–matter interactions; thus, they are being explored for various photonic and optoelectronic applications in a wide range of areas, including highly sensitive bio-sensing, subwavelength optics, photo-detectors, optical information processing, and data storage [1]. Conventional plasmonic materials commonly use noble metals, such as gold and silver, which can supply an abundant amount of free electrons for producing high-frequency plasmon resonances in passive and active plasmonic devices [2]. Unfortunately, these noble metal selves are hardly tunable, and inherent ohmic losses limit their usability in applications where such optical losses cannot be tolerated. In this context, all sorts of materials with metallic properties, beyond gold and silver, have been well researched for alternative plasmonic materials with the advantages of design flexibility, fabrication compatibility, and dynamic tunability, such as heavily-doped semiconductors, transparent conducting oxides (TCOs), and two-dimensional (2D) materials [3]. Among these alternative plasmonic materials, ultrathin 2D materials, such as graphene [4,5] and black phosphorus [6], display the capability of a plasmonic response with low damping rates and high confinements of light. Surface plasmons that are confined to the surface of 2D materials possess remarkable features, making them appealing alternatives to conventional metal-based plasmonic materials. However, due to their low carrier density (10^12^~10^14^ cm^-2^), the plasmonic response wavebands of these 2D materials are restricted to the terahertz (THz) or infrared regions [4,5,6], below those of the optical responses to many biologically interesting molecules. Two-dimensional materials with a higher carrier density and plasmonic response frequencies are highly coveted, as an emerging 2D flatland for biosensing, bioimaging, and theragnostic applications.

Borophene, a monolayer boron sheet, exhibits unique 2D metallic properties rarely found in other 2D materials. Compared to graphene and BP, borophene has a higher carrier density (~10^15^ cm^-2^) [7]. Theoretical studies have suggested that borophene can support a highly anisotropic and adjustable plasmonic response with low damping rates in a broad frequency range from the near-infrared to visible regions, thanks to its extremely high Dirac electron density [7]. With successive breakthroughs for 2D boron monolayer (i.e., borophene) experimental synthesis, either as freestanding atomic sheets via sonochemical liquid-phase exfoliation [8] or as continuous nanosheets on metals [9,10,11] and insulator [12] substrates through molecular beam epitaxy, borophene as a highly promising 2D material has sparked significant research interest, owing to its remarkable electrical and optical properties. Borophene is emerging as an extraordinary nanomaterial platform, substituting its predecessors in the fields of biomedical sensors, energy storage, theragnostics, and supercapacitors [13]. Particularly, borophene is very suitable for gas sensing applications due to the quite strong binding of the target gas molecules (such as CO, CO_2_, NO, NO_2_, NH_3_, and HCN, etc.) on its surface [14].

As a favorable plasmonic material, borophene is being extensively explored in the field of plasmonics. For instance, it has been revealed that borophene nanoribbons and nanopatches can support anisotropic localized surface plasmons with polarization-sensitive absorption and tunability in visible wavelengths [15]. A borophene ribbon array has been proposed to support plasmonic resonances, which can sense the local refractive index (RI) of the surrounding environment via spectral response [16]. Based on electrically tunable borophene plasmons, Feng et al. theoretically presented the active modulation of graphene electroabsorption in the optical communication waveband [17]. Employing an anisotropic borophene monolayer covered on a Si_3_N_4_ nano-hole array slab, Liu et al. achieved enhanced polarization-dependent absorption through critical coupling in visible light [18]. Moreover, the highly confined and tunable plasmonic modes in the borophene nanostructure can couple between different types of ones (i.e., localized plasmonic mode and guided plasmonic mode [19]), or with other resonant modes (magnetic polaritons [20] and Bloch surface wave [21]) and bring about many interesting phenomena, such as effective transmission modulation [22], plasmon-induced transparency (PIT) [19], and Rabi splitting [21]. These remarkable features enable us to actively govern the interactions between light and matter on a deep subwavelength scale and exploit innovative optical devices of remarkably small sizes. Therefore, it is imperative to delve deeper into the plasmonic behavior of borophene, as it could pave the way for the promising applications of borophene-based active nanophotonics in the near-infrared regime.

In this work, we proposed a borophene plasmonic hybrid system with a dynamically controllable PIT effect for high-performance broadband-tunable sensing in the near-infrared region (NIR). The PIT is realized by the strong coupling of dark and bright plasmonic modes, which originate from a localized surface plasmon (LSP) mode on sodium nanostrip gratings (SNGs) and a propagating borophene surface plasmon (BSP) mode in a continuous borophene monolayer (CBM) waveguide, respectively. Depending on electrically gating the borophene, the hybridization between these two plasmonic modes can be flexibly controlled and generates a tunable narrow PIT window in the broad absorption spectrum of the bright plasmonic mode. This hybrid plasmonic system exhibits a good RI-sensing performance associated with a wide range of dynamically adjustable wavebands by tuning the electron density of borophene. Our work reveals that borophene, as a favorable alternative to plasmonic materials, has great potential in active plasmonic sensors and can be applied in various plasmonic nano-devices.

## 2. Materials and Methods

**Figure 1:**
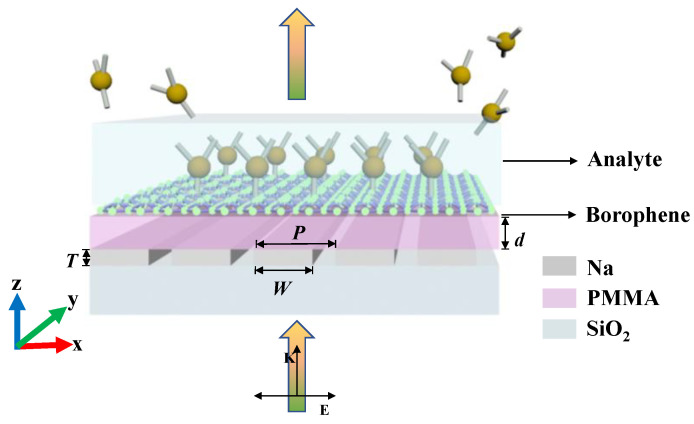
Schematic diagram of the proposed borophene-based hybrid sensing system.

The schematic view in Figure 1 depicts the borophene-based hybrid plasmonic structure in this work. It is a prime metal-insulator-metal (MIM) structure with a *χ*_3_-phase borophene monolayer and bottom sodium strips separated by a polymethyl methacrylate (PMMA) spacer with a thickness of *d* = 50 nm. Thanks to its superior light transmission and mechanical strength, PMMA is a popular polymer material for optical applications. The RI of PMMA polymer film was obtained from Ref. [23]. Polymers have extensive applications due to their easy manufacturing and compatibility, such as polymeric membranes for methyl orange dye degradation [24]. Herein, monolayer borophene can be synthesized on a metallic substrate and transferred into the hybrid system via a PMMA-based technique, which is popular and commonly used in the process of 2D material transfer [25]. In particular, for the designed structure, the PMMA polymer layer can act as an available layer rather than a sacrifice layer, which dispenses with PMMA removal and prevents any organic particle residues from affecting the quality of the borophene. The sodium grating, with a period *P* = 250 nm, width *W* = 180 nm, and height *T* = 10 nm, is arranged on a SiO_2_ substrate. The RI of the SiO_2_ substrate is assumed to be *n_sub_* = 1.45. The sensing interface is located on the top surface of the borophene monolayer, where gas or liquid analytes covered on it can be sensitively detected via resonant wavelength interrogation. Namely, the resonant wavelength of the PIT window will shift with changes in the analytes’ RIs. The much more detailed sensing mechanism will be elaborated in the following content.

Borophene, as a 2D material composed of boron atoms arranged in a hexagonal lattice, cannot exist in a perfect, defect-free form. Instead, it requires the presence of vacancies or missing atoms in its structure to become stable. Among several borophene polymorphs, three phases of borophene, *α*, *β*_12_, and *χ*_3_, have been subject to considerable scrutiny. Of them, *α* and *χ*_3_ possess pronounced crystallographic anisotropy in their crystal orientations. Nevertheless, *α* sheets are not experimentally stable due to a lack of vacancies [15]. The *χ*_3_ phase is constructed from more slender boron zigzag chains interspersed with arrays of holes. Herein, we chose *χ*_3_-phase borophene to theoretically investigate the PIT effect in the strongly coupled plasmonic system. Hereafter, the *χ*_3_ phase is used in this work. Certainly, all the forthcoming discussions can be applied to *α*- and *β*_12_-phase borophene as well.

Plasmon response is typically dependent on intraband transitions, which mainly refer to the responses of free carriers and are commonly modeled using the Drude equation. The metallic properties of monolayer borophene have been confirmed, owing to its significantly higher density of free electrons (i.e., Drude weight) as compared to other 2D materials [26,27]. Based on this, monolayer borophene in this study, without taking its thickness into account, is modeled as the surface current density, which is defined as the anisotropic conductivity *σ_jj_* based on the semiclassical Drude formula [15]:

(1) $$\sigma_{jj}(\omega )=\frac{iD_{j}}{\pi (\omega +i\tau^{-1})},D_{j}=\frac{\pi e^{2}n_{s}}{m_{j}}$$

where *j* stands for the borophene optical axis in the *x* or *y* crystallographic directions, *ω* is the angular frequency of the incident light, *τ* = 65 fs is the relaxation time of an electron, *D_j_* is the Drude weight, *n_s_* is the electron density, *e* is the electronic charge, and *m_j_* is the effective electron mass of the borophene. In this work, *χ*_3_-phase borophene was used with *m_x_* = 1.4*m*_0_ and *m_y_* = 3.4*m*_0_, *m*_0_ as the standard electron masses. 

Sodium, being an alkali metal, has been widely considered as an excellent material for plasmonics due to its low intraband damping rate [28]. In particular, its plasmonic response waveband is located around 1.5 μm [29], which matches with that of the borophene monolayer waveguide. Therefore, alkali metal sodium is chosen as the grating material. The permittivity of sodium can be described by using a Drude–Lorentz model [28]:

(2) $$\varepsilon_{m}=\varepsilon_{b}-\frac{\omega_{p}{}^{2}}{\omega^{2}+i\omega \gamma_{p}}+\frac{f_{1}\omega_{1}{}^{2}}{\omega_{1}{}^{2}-\omega^{2}-i\omega \gamma_{1}}$$

where the polarization response from the core electrons *ε_b_* = 0.500, the bulk plasma frequency *ω_p_* = 5.414 eV, the resonant frequency and amplitude of the inter-band transition *ω*_1_ = 2.945 eV and *f*_1_ = 0.280, respectively, the related interband damping rate *γ*_1_ = 2.706 eV, and the Drude damping rate *γ_p_* = 0.010 eV. 

Numerical simulations are performed with the finite difference time domain method to investigate the PIT mechanism in the hybrid system. Since the structure is homogeneous along the ± *y* directions, the 2D numerical simulation is implemented in the *x*-*z* domain of the structure. In the simulations, a periodic boundary condition is applied in the horizontal direction, while a perfectly matched layer (PML) is imposed in the vertical direction at two ends of the computational space to achieve the absorbing boundary conditions. A plane wave source with *x*-polarization is illuminated from the bottom of the structure to perform the far-field excitation, as presented in Figure 1, and a monitor is placed at the top of the structure to detect the transmission spectra. Non-uniform mesh is employed to mesh the simulation region. To ensure accuracy, a high-density mesh with a size of 0.03 nm/div is used within the borophene monolayer region, while the grid size gradually increases beyond this region. The plasmonic response in the anisotropic borophene monolayer is similar in both the *x* and *y* directions—only the PIT window occurs at different resonant wavelengths. For the borophene with same electron density, the PIT response wavelength is longer along the *y* direction compared to that along the *x* direction. This can be attributed to the fact that the electron mass of the borophene along the *y* direction is larger than that along the *x* direction. Given this, all the forthcoming discussions will solely focus on the case of borophene in the *x* direction, to avoid any duplication and repetition.

## 3. Results and Discussion

### 3.1. Controllable Coupling Mechanism

PIT is an analogue of electromagnetically induced transparency (EIT) in plasmonic metamaterial systems, which can give rise to a relatively narrow transparency window in a broad absorption spectrum [30]. In general, the PIT phenomenon can be achieved by either inducing direct destructive interference between a bright mode and dark mode [31], or by introducing a detuning mechanism for two bright modes via the symmetry breaking of plasmonic metamaterial structures [32]. In the proposed structure, a PIT effect occurs through the longitudinal coupling between the LSP mode on the sodium nanostrips and the BSP mode in the CBM waveguide, which are considered as the bright mode and dark mode, respectively. 

**Figure 2:**
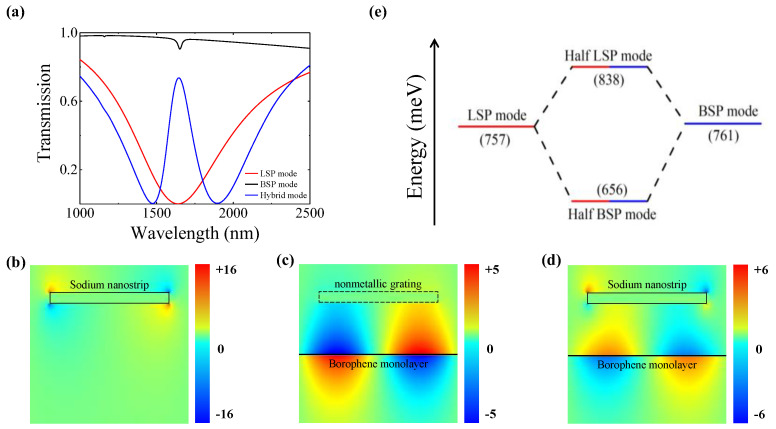
(**a**) Transmission spectra of the LSP, BSP, and hybrid modes at borophene electron density of *n_s_* = 9.7 × 10^19^ m^−2^. The corresponding *E_y_* distributions of the LSP mode on bare SNGs (**b**), BSP mode in CBM waveguide with Si grating (**c**), and hybrid mode in hybrid structure at PIT peak (**d**). (**e**) Energy diagram corresponds to the LSP and BSP modes hybridization.

For the purpose of elaborating the underlying physical mechanism of this PIT system, the transmission spectrum was first calculated for the bare SNGs without CBM in the structure. As depicted by the red line in Figure 2a, a deep resonance dip at a wavelength of 1.65 μm is observed in the transmission spectrum, which results from the LSP mode’s direct excitation on the bare SNGs via the TM-polarized incident light. Serving as a bright plasmonic mode, the LSP mode has a broad enough resonance dip due to strong radiative loss. The corresponding electric field distribution *E_y_* for the LSP mode at 1.65 μm is provided in Figure 2b. As a localized plasmonic mode, the excited electric field mainly concentrates at four corners of the sodium nanostrip, rather than on its surface, as shown in Figure 2b. Moreover, the resonant wavelength of the LSP mode is independent of the nanostrip grating material and is determined by the grating constant and surrounding RI.

On the other hand, the propagating BSP mode in the CBM waveguide, serving as a dark mode, cannot be directly excited but requires phase matching with the assistance of SNGs. As the black line shown in Figure 2a, the transmission spectrum for the BSP mode is excited at a resonant wavelength position (~1.65 μm) that is nearly identical to that of the LSP mode. However, the transmission dip of the BSP mode is considerably smaller in comparison. As shown in the mode profile illustrated in Figure 2c, the electric field distribution *E_y_* for the BSP mode is relatively weakly and symmetrically concentrated around the borophene monolayer. The BSP mode’s electromagnetic energy experiences a minor dissipation on the ultrathin borophene monolayer, as a result of ohmic loss, which is reflected in a shallow dip in the high transmittance. Note that the BSP mode is unable to be directly excited in the CBM waveguide by incident light because of its large mismatch in wavevectors with light in free space. In order to address this wavevector mismatch, the simulations utilized nonmetallic grating with the same period as that of the SNGs to prompt BSP mode excitation under the phase-matching condition [33]:

(3) $$Re(\beta (\omega ))=\frac{\omega_{0}}{c}sin\theta +\frac{2\pi }{p}$$

where *p* is the grating period, *c* is the speed of vacuum light, and *ω*_0_/*c* equals the free space wavevector. The incident angle *θ* is set as 0° in this work. The wavevector *β*(*ω*) of the BSP mode in the CBM waveguide can be derived by solving the dispersion equation for the guided mode [34]:

(4) $$\frac{n_{p}{}^{2}}{\sqrt{\beta {\left(\omega \right)}^{2}-n_{p}{}^{2}k_{0}{}^{2}}}+\frac{n_{a}{}^{2}}{\sqrt{\beta {\left(\omega \right)}^{2}-n_{a}{}^{2}k_{0}{}^{2}}}=-\frac{i\sigma_{jj}}{ck_{0}\varepsilon_{0}}$$

where *k*_0_ = 2π/*λ* is the vacuum wavevector and *n_p_* and *n_a_* are the RIs of the spacer polymer and analyte, respectively.

By combining the SNGs with the CBM waveguide into the proposed hybrid plasmonic system, a strong coupling between the LSP mode with a broad dip and the BSP mode as a shallow and narrow dip can occur in the system when they are brought into resonance simultaneously, thus leading to the emergence of a towering PIT transparency window in the transmission spectrum (blue line in Figure 2a). The PIT window position is sensitive to the analyte RI due to the resonant wavelength of the BSP mode shifting with a varying analyte RI, which is deducible from Equation (4). As shown in Figure 2d, the electric field distribution *E_y_* for the hybrid mode at the wavelength of the PIT peak position indicates that the coupling of the two resonance modes results in a destructive interference and suppresses the LSP mode’s intensity on the SNGs to a low magnitude, which also can be clearly observed from the comparison of the field distributions in Figure 2b–d. This is the primary reason that the transmission is enhanced to generate a narrow PIT transparency window from a broad resonance dip. Here, the borophene electron doping density is set to be *n_s_* = 9.7 × 10^19^ m^−2^. 

Akin to the EIT phenomena in three-level atomic systems, the coupling process described above can be elucidated using the standard coupled oscillator model (COM), which treats the LSP mode and BSP mode as classical oscillators. The energies of hybrid modes can be simplified into a two-oscillator model [35]:

(5) $$E_{L}-E_{H}=\frac{{\left(ћ\Omega \right)}^{2}}{4(E_{B}-E_{H})}$$

Here, *Ω* refers to the coupling frequency, which is utilized to assess the level of coupling between the LSP mode and BSP mode. *E_H_* is the hybrid mode energy. *E_L_* and *E_B_* are the energies of the LSP mode and BSP mode, respectively, which are related to their resonant frequencies. Upon solving Equation (5), two solutions for *E_H_*, which correspond to the energies of the hybrid modes, can be obtained. Further, based on the correspondence between the energy and frequency, the resonant frequencies of two new transmission dips for the hybrid modes can be deduced and simplified as [36]

(6) $$f_{H}=\frac{f_{L}+f_{B}\pm \sqrt{{\left(f_{L}-f_{B}\right)}^{2}+\Omega^{2}}}{2}$$

where *f_L_*, *f_B_*, and *f_H_* are the resonant frequencies of the LSP mode, BSP mode, and two hybrid modes, respectively.

The coupling process refers to the hybridization for the LSP mode and BSP mode, which is accompanied by an energy exchange. As the electron density of the borophene *n_s_* = 9.7 × 10^19^ m^−2^, the plasmonic resonance frequencies for the two modes are nearly same. In other words, the coupling between the LSP mode and BSP mode occurs at zero detuning of the resonant wavelength, resulting in a strong exchange of light energy between the top SNGs and bottom CBM surface via mode hybridization. Figure 2e provides the energy diagram for this mode hybridization, which reveals that these two modes are equally blended in the hybrid modes due to the identical resonant frequencies of the BSP and LSP modes. As illustrated in Figure 2e, the hybrid modes exist in a state of an intermediate nature, which exhibits the feature of half the LSP mode and half the BSP mode.

**Figure 3:**
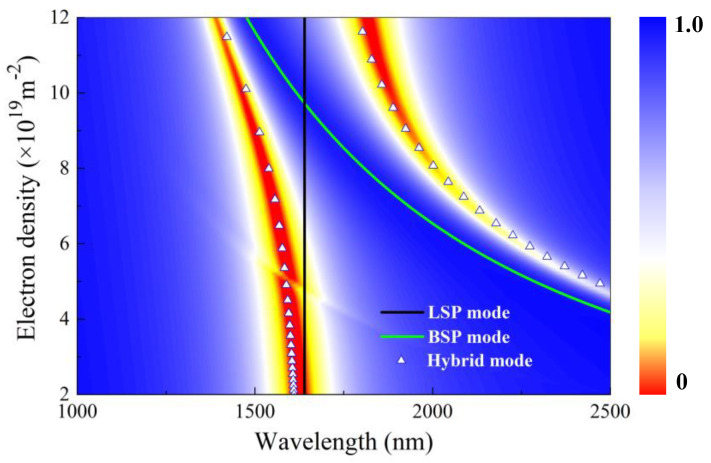
Transmission spectral contour map for hybrid modes as electron density *n_s_* varied from 2 × 10^19^ m^−2^ to 12 × 10^19^ m^−2^. The lines in the diagram represent the resonant wavelength of LSP mode (black line), BSP mode (green line), and hybrid mode (small white triangles), respectively.

According to Equations (3)–(6), the dispersion relations of two hybrid modes (white solid triangles), LSP mode (black line), and BSP mode (green line) are presented in Figure 3. Here, the resonant wavelength of the LSP mode remains unchanged. As shown in Figure 3, the resonant wavelength of the BSP mode and PIT peak wavelength both blueshift by increasing the electron density of the borophene *n_s_* from 2 × 10^19^ m^−2^ to 12 × 10^19^ m^−2^. Apparently, at a borophene electron density of *n_s_* = 9.7 × 10^19^ m^−2^, the BSP mode and LSP mode dispersion curves are found to cross each other. In contrast, the dispersion curves of the two hybrid modes without any intersection exhibit an anti-crossing behavior with an energy gap, indicating the typical Rabi splitting phenomenon [36]. The analytical results are in good agreement with the numerical results, confirming the occurrence of hybridization between the LSP mode and BSP mode. As the electron density is significantly higher or lower than *n_s_* = 9.7 × 10^19^ m^−2^, the LSP mode and BSP mode are widely separated, and they demonstrate independent behavior.

**Figure 4:**
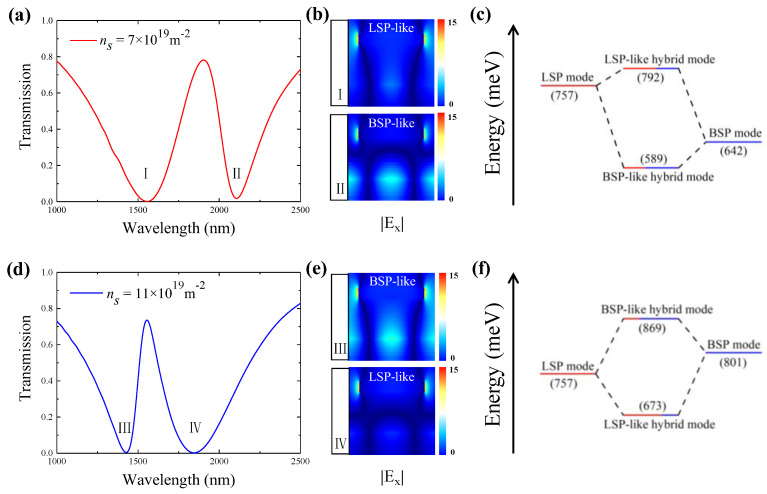
The PIT transmission spectra at electron density of *n_s_* = 7 × 10^19^ m^−2^ (**a**) and *n_s_* = 11 × 10^19^ m^−2^ (**d**). The corresponding electric field distributions |*E*_x_| at the transmission dips as electron density of *n_s_* = 7 × 10^19^ m^−2^ (**b**) and *n_s_* = 11 × 10^19^ m^−2^ (**e**). The energy diagrams (**c**,**f**) correspond to the cases in (**a**,**d**), respectively.

The energy exchange between the hybrid modes is further analyzed to gain a deeper insight into the coupling process between the LSP and BSP modes. Herein, we take two distinct electron densities as representative illustrations, i.e., *n_s_* = 7 × 10^19^ m^−2^ and *n_s_* = 11 × 10^19^ m^−2^. Their transmission spectra are purposely provided in Figure 4a,d for a more intuitive view. Compared to the case of *n_s_* = 9.7 × 10^19^ m^−2^, the PIT peak wavelengths for *n_s_* = 7 × 10^19^ m^−2^ and *n_s_* = 11 × 10^19^ m^−2^ redshift to 1834.1 nm and blueshift to 1494.8 nm, respectively. 

At a lower borophene electron density of 7 × 10^19^ m^−2^, the transmission spectrum has a broad dip (the left dip marked with I in Figure 4a) and a narrow dip (the right dip marked with II in Figure 4a), which correspond to LSP-like and BSP-like hybrid modes, respectively. This can be verified by the electric field distributions |*E*_x_| depicted in Figure 4b. As shown in Figure 4b, the LSP-like hybrid mode (labeled as I in Figure 4b) acquires more LSP mode features. Specifically, the electric field of the mode is localized at the two side facets of the SNG, which are distinctive features of the LSP mode, while the characteristics of the BSP-like hybrid mode (labeled as II in Figure 4b), resembling more of a BSP mode, are evidenced by its relatively narrower resonant dip and stronger BSP mode feature in the mode field pattern. Figure 4c shows the energy diagram of the hybrid modes for this case. As indicated by the diagram, the hybridization of the LSP and BSP modes with moderate energy levels produces two hybrid modes with distinct energy levels: the LSP-like mode (at a shorter wavelength) with higher energy and the BSP-like mode (at a longer wavelength) with lower energy. 

Differently, at a higher borophene electron density of 11 × 10^19^ m^−2^, the LSP-like hybrid mode appears at a longer wavelength, while the BSP-like hybrid mode emerges at a shorter wavelength. The dip associated with the BSP-like hybrid mode (labelled as Ⅲ in Figure 4d) moves to the left of the resonant dip of the LSP-like hybrid mode, labelled as Ⅳ in Figure 4d. Similar to the above discussion for the case of *n_s_* =7 × 10^19^ m^−2^, the corresponding mode field patterns in Figure 4e confirm these results. The energy diagram shown in Figure 4f illustrates that the higher energy of the BSP mode, relative to the LSP mode, gives rise for the upper hybrid mode to transform into a BSP-like hybrid mode, rather than an LSP-like hybrid mode.

### 3.2. Broadband Tunable Sensitivity Characteristics

In general, unlike physical and electrochemical sensors, optical sensors tend to respond more strongly to changes in RI through spectral variations. Typically, the optical-sensing mechanism depends on the degree of resonant wavelength alteration triggered by fluctuations in the RI. Despite advancements in photonic technologies that have enhanced the sensing capabilities of these optical sensors, the absence of active tunability in static sensors hinders their operating frequency and the range of analytes they can detect. Efforts have been made to develop RI-sensing devices with a dynamic tunability, such as frequency-tunable metamaterials using mechanically stretchable substrates or bulk Dirac semimetals with adjustable Fermi levels [37,38]. Despite these advances, slow regulating speeds and complex configurations still pose challenges for their application prospects in RI sensing.

**Figure 5:**
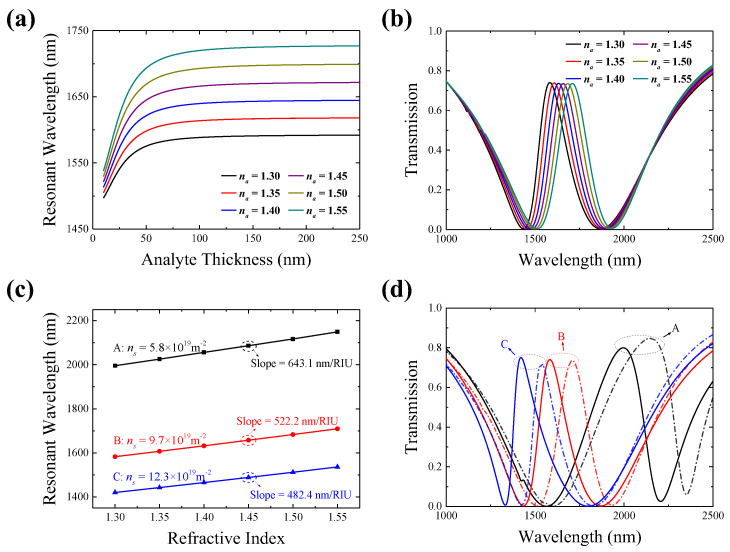
(**a**) The resonant wavelengths with different thicknesses of analyte layers varying from 10 nm to 250 nm. (**b**) Taking *n_s_* = 9.7 × 10^19^ m^−2^ as an example, the transmission spectra at different RIs of analyte. (**c**) The PIT resonant wavelength shift for the different RIs of analytes at *n_s_* = 5.8 × 10^19^ m^−2^, *n_s_* = 9.7 × 10^19^ m^−2^, and *n_s_* = 12.3 × 10^19^ m^−2^, respectively. (**d**) The transmission spectra evolution corresponds to the three cases in (**c**) as RI changes from 1.30 to 1.55.

In the proposed sensing system, one can measure the analyte RI variation via monitoring the PIT windows’ shift in the transmission spectra of the targeted analytes covering the borophene monolayer. It is noteworthy that the analyte covered on the borophene should exceed a certain thickness, considering that the thickness of the overthin analyte layer would disturb the resonant wavelength. To eliminate the possibility of sensing inaccuracies stemming from variations in this analyte thickness, we commence our study by examining the interrelationship between the resonant wavelength and analyte thickness. As shown in Figure 5a, the resonant wavelength of the analytes undergoes a redshift as the thickness of the analyte layer increases from 10 nm to 250 nm. After the thickness of the analyte layer surpasses 200 nm, all the resonant wavelengths tend to maintain unchanged values. The analyte thickness (>200 nm) does not influence the resonant wavelengths, because the evanescent electric field of the SP modes penetrating into the analyte layer from the sensing surface is mainly within a distance of the order of 200 nm [39]. To eliminate any potential spectral shifts that may arise from alterations in the thickness of the analyte layer, it is advisable to ensure that the analyte layer thickness exceeds the penetration depth of the SP evanescent waves.

The sensitivity of an optical sensor is one of the important criteria to reflect its detection capability. The shift amount of a PIT peak can be considered as an indicator for RI sensing. Here, the RI sensitivity *S*, defined as the PIT resonant wavelength shift per unit RI change of the analyte, can be expressed as

(7) $$S=\frac{\Delta \lambda }{\Delta n_{a}}$$

where Δ*λ* is the PIT peak wavelength shift amount with units of nm and Δ*n_a_* denotes the corresponding variation in the analyte RI.

Figure 5b provides the transmission spectra evolution with the analyte RI variation in the case of a borophene electron density *n_s_* = 9.7 × 10^19^ m^−2^ and the other structure parameters being unchanged. As indicated in Figure 5b, all the transmission spectra maintain consistent spectral lineshapes with a stable PIT peak intensity. When the analyte RI increases, the PIT peaks shift towards longer wavelengths. Specifically, as the analyte RI varies from 1.30 to 1.55, the PIT peak wavelength undergoes a redshift of 130.5 nm, shifting from 1581.7 nm to 1712.2 nm. As mentioned above, the resonant condition for the BSP mode relies on the ambient RI due to the dependency of its wavevector on the RI, which leads to the PIT window shifting correspondingly with RI changes. In addition, Equation (4) clearly indicates that the wavevector of the BSP mode is also determined by the conductivity of the borophene. Considering these two factors, tunable sensing can be achieved by actively adjusting the electron density of the borophene. This can be effectively realized through external electrical stimuli, as exemplified in Ref. [17].

In order to assess the tunable sensing capabilities of the proposed sensing system in a quantitative manner, Figure 5c displays the spectral resonance positions as a function of the varying RI of the analyte at three representative borophene electron densities of *n_s_* = 5.8 × 10^19^ m^−2^, *n_s_* = 9.7 × 10^19^ m^−2^, and *n_s_* = 12.3 × 10^19^ m^−2^. The PIT peak wavelengths are plotted against the analyte RI, and linear fitting is performed to illustrate the trend of the wavelength shift with respect to the RI, as depicted by the data points and fitting lines in Figure 5c. This provides clear evidence of a robust linear relationship between the PIT peak wavelength shift and the analytical RI variation. Therefore, the detection of RI changes can be accomplished by observing the PIT peak wavelength shifts. The fitted line slopes in Figure 5c serve as the RI sensitivities of the sensing system. Quantitatively, the RI sensing sensitivities reach 643.1 nm/RIU, 522.2 nm/RIU, and 482.4 nm/RIU for the three different borophene electron densities of *n_s_* = 5.8 × 10^19^ m^−2^, *n_s_* = 9.7 × 10^19^ m^−2^, and *n_s_* = 12.3 × 10^19^ m^−2^, respectively. On the other hand, as illustrated in Figure 5c, the resonant wavelength can be tuned from 1420 nm to 2150 nm as the borophene electron density *n_s_* is adjusted from 12.3 × 10^19^ m^−2^ to 5.8 × 10^19^ m^−2^. Thus, the proposed sensing system exhibits a good RI-sensing performance associated with an ability to operate in a wide range of wavelengths in a tunable manner, which is favorable for different working band requirements and the object detection of analyte categories. Moreover, the detailed transmission spectra evolution is offered in Figure 5d, when the analyte RIs increase from 1.30 to 1.55 under three different borophene electron densities. As shown in Figure 5d, the PIT windows of the transmission spectra for the different electron densities consistently have a high transmittance and obvious resonant wavelength variation. The RI sensing is more sensitive at a lower borophene electron density. However, the symmetry of the PIT window weakens due to the deviation of the PIT peak from the resonant wavelength position of the LSP mode.

**Figure 6:**
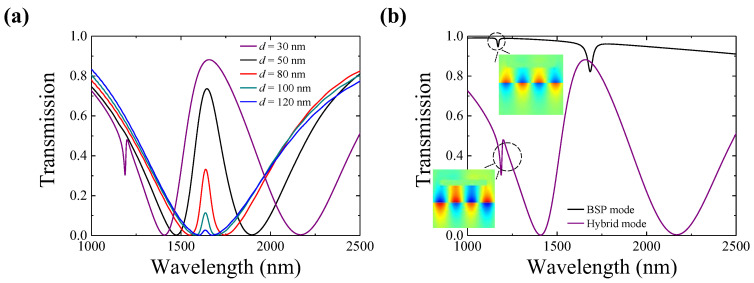
(**a**) Transmission spectrum evolution as the spacer layer thickness increases from 30 nm to 120 nm. (**b**) BSP mode (black line) and hybrid mode (purple line) spectra when the thickness of spacer value is 30 nm. The insets in Figure 6b show the electric field distributions *Ey* for the newly emerged 3rd order BSP mode and Fano resonance mode.

Finally, we also investigate the influence of the PMMA spacer layer thickness between the CBM and SNGs on the coupling strength, which is crucial to the PIT peak amplitude in this longitudinal coupling system. As illustrated in Figure 6a, the PIT peak within the transparency window significantly decreases as the thickness of the spacer layer gradually increases, eventually leading to the disappearance of the peak. In addition, a sharp transparency sub-band with a typical Fano resonance nature appears at the left flank of the PIT window when the spacer layer thickness decreases to 30 nm, and the amplitude of the sub-band will increase as the spacer layer thickness becomes smaller. Taking the spacer layer thickness of 30 nm as an example, the transmission spectra of the BSP mode and hybrid mode are plotted together in Figure 6b. As shown in Figure 6b, a weak absorption dip is observed at 1.17 µm in the BSP mode transmission spectrum, corresponding to the third-order BSP mode resonance in the CBM waveguide. The destructive interaction between the broadband LSP mode on the SNGs and the narrowband higher-order BSP mode in the CBM waveguide leads to Fano resonance in the transmission spectrum of the hybrid system. The electric field distributions at 1.17 µm for the third-order BSP mode and the Fano resonance mode in the insets in Figure 6b can also confirm these analysis results.

## 4. Conclusions

In conclusion, we proposed and theoretically investigated a borophene-based PIT hybrid system and discussed its high-sensitivity and broadband-tunable RI-sensing ability in near-infrared wavelengths. The PIT effect was realized via the strong longitudinal coupling between the LSP and BSP modes. This coupling strength could be significantly impacted by the thickness of the PMMA spacer layer, which determined the detection intensity of the PIT transparency window. A coupled two-oscillator model was employed to quantitatively describe the observed LSP–BSP coupling, which showed a good agreement with the simulation results. The PIT window could be flexibly tuned by dynamically adjusting the electron density of the borophene. By actively varying the borophene electron density to drive the system into the strong coupling regime, the Rabi splitting phenomenon appeared. Furthermore, our investigations revealed that the PIT window also displayed a dependence on ambient RIs. Owing to these, the hybrid system exhibited a good RI-sensing sensitivity of 612 nm/RIU, associated with a wide range of dynamically tunable working wavebands (1420–2150 nm), which would allow one to actively operate the sensing system to selectively work within desired wavebands. This work uncovered the potential applications of borophene plasmon in advanced point-of-care biomedical diagnostics, and broadens the scope of possibilities for designing various borophene-based active optoelectronic and photonic nanodevices operating in the NIR range. 

## Data Availability

The data presented in this study are available upon reasonable request from the corresponding authors.
